# Predicting prognosis in colorectal cancer patients with curative resection using albumin, lymphocyte count and RAS mutations

**DOI:** 10.1038/s41598-024-65457-8

**Published:** 2024-06-23

**Authors:** Toshiya Miyata, Tamuro Hayama, Tsuyoshi Ozawa, Keijiro Nozawa, Takeyuki Misawa, Takeo Fukagawa

**Affiliations:** 1 Department of Surgery, Teikyo University School of Medicine, 2-11-1 Kaga, Itabashi-ku, Tokyo, 173-0003 Japan; 2 Department of Surgical Oncology, Graduate School of Medicine, The University of Tokyo, Tokyo, Japan

**Keywords:** Lymphocyte, Albumin, *RAS*, Prognostic factor, Colorectal cancer, Cancer, Gastroenterology, Oncology

## Abstract

Colorectal cancer (CRC) poses a significant global health challenge, demanding reliable prognostic tools to guide treatment decisions. This study introduces a novel prognostic scoring system, the albumin-total lymphocyte count-*RAS* index (ALRI), integrating serum albumin, lymphocyte count, and *RAS* gene mutations. A cohort of 445 stage I–III CRC patients undergoing curative resection was analyzed, revealing ALRI's association with clinicopathological factors, including age, tumor location, and invasion depth. The ALRI demonstrated superior prognostic value, with a cutoff value of 2 distinguishing high and low-risk groups. The high-ALRI group exhibited elevated rates of recurrence. Univariate and multivariate analyses identified ALRI as an independent predictor for both 5 year recurrence-free survival (RFS) and overall survival (OS). Kaplan–Meier curves illustrated significant differences in RFS and OS between high and low-ALRI groups, emphasizing ALRI's potential as a prognostic marker. Importantly, ALRI outperformed existing nutritional indices, such as controlling nutritional status and neutrophil-to-lymphocyte ratio, in predicting overall survival. The study underscores the comprehensive insight provided by ALRI, combining inflammatory, nutritional, and genetic information for robust prognostication in CRC patients. This user-friendly tool demonstrates promise for preoperative prognosis and personalized treatment strategies, emphasizing the crucial role of inflammation and nutrition in CRC outcomes.

## Introduction

Colorectal cancer (CRC) constitutes a significant global health challenge, contributing significantly to cancer-related morbidity and mortality^1^. Although surgery stands as the foremost treatment modality, the spectrum of postoperative complications and long-term prognoses varies considerably. The identification of dependable prognostic factors holds paramount importance in guiding treatment strategies and enhancing patient outcomes.

In recent years, there has been a widespread acknowledgment that the prognosis of cancer patients is intricately connected to either the characteristics of the tumor or numerous host-related factors^2,3^. Notably, tumor characteristics, specifically the presence of *KRAS* G12V or G12C mutations, have been reported to correlate with a poorer prognosis in CRC^4,5^. Turning to host-related factors, there is a growing emphasis on understanding the impact of the inflammatory state on the prognosis of patients with malignant tumors. Inflammation associated with cancer plays a role in tumor proliferation, the promotion of angiogenesis, and metastasis.

Various systemic inflammatory markers, including the neutrophil-to-lymphocyte ratio (NLR), lymphocyte-to-monocyte ratio (LMR), platelet-to-lymphocyte ratio (PLR), and others, have been linked to the prognosis of CRC patients^6–8^. Furthermore, accumulating evidence continues to establish an association between nutritional status and the short-term and long-term prognosis of CRC^9^. Notably, several nutritional indicators, such as serum albumin levels and total cholesterol, have been scrutinized for their independent correlation with survival outcomes in CRC^10^.

Various simplified scoring systems, incorporating one or more inflammatory or nutritional parameters, such as the geriatric nutritional risk index (GNRI), controlling nutritional status (CONUT), and systemic inflammatory score (SIS), are widely employed to predict outcomes^11–13^. Nevertheless, these predictive factors remain insufficient due to their limited representation of the overall condition of cancer patients. We aim to offer a comprehensive perspective on cancer prognosis by incorporating nutritional indices, inflammatory responses, and genetic mutations as key factors.

Consequently, we developed a novel scoring system to assess the prognosis of CRC, utilizing serum albumin, lymphocyte count, and *RAS* gene mutations. This system, named the albumin- total lymphocyte count-*RAS* index (ALRI), comprises three parameters: serum albumin, lymphocyte count, and *RAS* gene mutations. ALRI offers a comprehensive reflection of the patient’s inflammatory and nutritional status, as well as genetic information about the cancer. In this study, we examined the utility of the ALRI scoring system that we have developed.

## Results

### Determination of cut-off values

**Figure 1:**
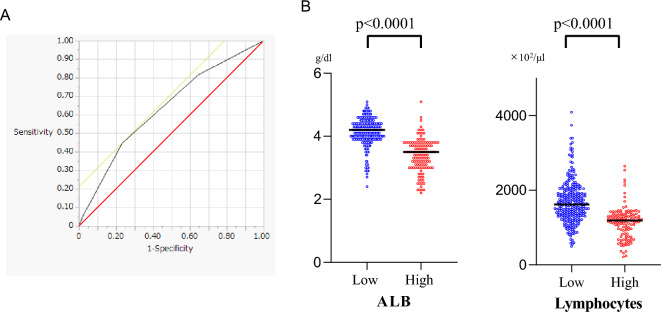
(**A**) The receiver operating characteristics curve of ALRI status. (**B**) Albumin, total lymphocyte count scattergraphs by ALRI.

The receiver operating characteristics (ROC) curve analysis results indicated that the most appropriate cutoff value for the ALRI was 2 (Fig. 1A). Area under the curve is 64, 57. All patients were categorized into the high ALRI score group (score ≥ 2; n = 319, 71.7%) or low ALRI group (score = 0 or 1; n = 126, 28.3%).

### Association between the ALRI and clinical characteristics

Among the 445 patients, there were 262 males and 183 females, with a mean age of 67.5 years (ranging from 22 to 94 years). According to the ALRI system, 319 patients (71.7%) fell into the low group (ALRI 0:140 or ALRI 1:179), while 126 patients (28.3%) were in the high group (ALRI 2:112 or ALRI 3:14). Based on the eighth version of the UICC/AJCC TNM classification^14^, 104 patients (23.3%) were categorized as stage I, 184 (41.3%) as stage II, and 157 (35.3%) as stage III. Over a median follow-up period of 41.5 months (ranging from 1 to 60 months), there were 46 patient deaths, accounting for 10.3% of the total cohort.

### ALB, total lymphocyte count scattergraph by ALRI

The average values for low ALRI were observed to be ALB: 4.1 and Lymphocytes: 1694, while for high ALRI, the values were 3.4 and 1140, respectively (Fig. 1B). ALRI is a quantification of serum albumin level, lymphocyte count and *RAS* mutations. Thus, there were significant differences in serum albumin level and lymphocytes between the high ALRI group and low ALRI group. It’s noteworthy that none of the individuals in the low-cholesterol group were using cholesterol medication.

### Associations of ALRI quality with clinicopathological factors

Table 1 outlines the correlation between ALRI levels and various clinicopathological factors such as age, gender, tumor location, pathological type, depth of tumor invasion, lymph node metastasis, lymph/venous invasion, CEA level, CA19-9 level and adjuvant chemotherapy. Significantly, ALRI levels demonstrated correlation with tumor location (p = 0.0001), pathological type (p = 0.0028), lymph invasion (p = 0.0167), venous invasion (p = 0.0167), depth of tumor invasion (p < 0.0001), CEA level (p = 0.0063), and CA19-9 level (p = 0.0035).

**Table 1:** The relationship between ALRI status and clinicopathological factors in the colorectal cancer patients.

Variables	High-ALRI group (n = 126)	Low-ALRI group (n = 319)	p-value
Age, yrs; ≤ 67/ > 67	38 (30.2%)/88 (69.8%)	172 (53.9%)/147 (46.1%)	0.0001
Males/females	75 (59.0%)/51 (41.0%)	187 (58.6%)/132 (41.3%)	0.8150
Tumor location, right side/left side	59 (46.8%)/67 (53.2%)	84 (26.3%)/235 (73.7%)	0.0001
Histology, well or moderate/others	105 (17.5%)/21 (82.4%)	291 (10.4%)/28 (89.6%)	0.0028
Depth of tumor invasion, T1–T2/T3–T4	20 (15.9%)/106 (84.1%)	123 (38.6%)/196 (61.4%)	0.0001
Lymph node metastasis, − / +	76 (60.3%)/50 (39.7%)	212 (66.5%)/107 (33.5%)	0.2277
Lymph invasion, − / +	53 (42.1%)/73 (57.4%)	201 (63.0%)/118 (37.0%)	0.0001
Venous invasion, − / +	28 (22.2%)/98 (77.8%)	108 (33.9%)/211 (66.1%)	0.0167
CEA level, high/normal	52 (41.3%)/74 (58.7%)	87 (27.4%)/231 (72.6%)	0.0063
CA19-9 level, high/normal	30 (23.8%)/96 (76.2%)	39 (12.3%)/279 (87.7%)	0.0035
Adjuvant chemotherapy, − / +	95 (24.8%)/31 (75.2%)	228 (71.9%)/89 (28.1%)	0.4034

*ALRI* albumin-lymphocyte-RAS index.

### Survival analysis of CRC patients based on their ALRI values

The survival analysis of CRC patients, encompassing a total of 445 individuals, was conducted over a median period of 1645 days (with a range of 10 to 2800 days). Among the cohort, 85 patients (19.1%) experienced disease recurrence. Within this subgroup, metastases were identified as follows: liver metastases in 27 patients (31.7%), lung metastases in 22 patients (25.9%), peritoneal carcinomatosis in 10 patients (11.8%), local recurrence in 8 patients (9.4%), para-aortic lymph node involvement in another 8 patients (9.4%), and the remaining 10 patients (11.8%) exhibited other forms of recurrence.

### Univariable and multivariable analyses of 5 year RFS (recurrence-free survival) and OS (overall survival)

We investigated the correlation between ALRI levels, clinicopathological factors, and the 5 year RFS rate in 445 patients. The cohort was divided into the low-ALRI group (ALRI ≤ 1, n = 319, 71.7%) and the high-ALRI group (ALRI > 1, n = 126, 28.3%). Univariate survival analyses presented in Table 2 indicated several factors significantly associated with a diminished 5 year RFS rate. These factors encompassed ALRI, histology, lymph invasion, pT category, pN category, preoperative CEA level, and CA19-9 level. Conversely, age, gender, tumor location, and venous invasion did not exhibit a significant association with the 5 year RFS rate. In determining independent prognostic factors for 5 year RFS, a multivariate analysis identified ALRI level, lymph invasion, pT category, pN category, and preoperative CEA level as independent prognostic factors (refer to Table 2).

**Table 2:** The univariate and multivariate analysis of prognostic factors for 5 year RFS.

Variables	Univariate			Multivariate		
	HR	95% CI	p-value	HR	95% CI	p-value
Age, yrs; ≤ 66, > 66	1.40	0.943–2.071	0.095			
Males/females	1.01	0.651–1.566	0.965			
Tumor location, right side/left side	1.19	0.791–1.778	0.411			
Histology, well or moderate/others	1.68	0.937–3.001	0.044			
Depth of tumor invasion, T1–T2/T3–T4	3.05	1.646–5.640	0.0004	2.34	1.252–4.383	0.007
Lymph node metastasis, − / +	1.74	1.141–2.659	0.0101	2.11	1.366–3.258	0.0007
Lymph invasion, − / +	1.96	1.263–3.042	0.0027	1.70	1.079–2.665	0.022
Venous invasion, − / +	1.52	0.889–2.597	0.1270			
CEA level, normal /high	2.01	1.343–3.016	0.0007	1.87	1.238–2.830	0.0030
CA19-9 level, normal /high	1.92	1.220–3.037	0.005	1.45	0.914–2.305	0.1221
ALRI	2.48	1.682–3.663	< 0.0001	1.84	1.231–2.751	0.0034

*ALRI* albumin-lymphocyte-RAS index.

Table 3 summarizes the results obtained from both univariate and multivariate analyses pertaining to the 5 year OS rate of patients. Univariate analyses revealed a significant association between 5 year OS and various factors, including pT category, pN category, lymph invasion, preoperative CEA level, and SIRI level. However, in the multivariate analyses specifically targeting 5 year OS, only the pN category, preoperative CEA level, and ALRI level emerged as independent predictive factors.

**Table 3:** The univariate and multivariate analysis of prognostic factors for 5 year OS.

Variables	Univariate			Multivariate		
	HR	95% CI	p-value	HR	95% CI	p-value
Age, yrs; ≤ 67, > 67	1.24	0.684–2.192	0.496			
Males/females	1.21	0.684–2.192	0.542			
Tumor location, right side/left side	1.32	0.414–1.381	0.363			
Histology, well or moderate/others	2.05	0.986–4.272	0.054			
Depth of tumor invasion, T1–T2/T3–T4	2.54	1.116–5.765	0.026	1.52	0.647–3.555	0.338
Lymph node metastasis, − / +	2.12	1.168–3.834	0.014	1.97	1.040–3.721	0.038
Lymph invasion, − / +	2.45	1.315–4.580	0.005	1.51	0.771–2.955	0..229
Venous invasion, − / +	1.76	0.806–3.863	0.156			
CEA level, normal /high	2.49	1.364–4.553	0.003	2.38	1.310–4.320	0.0044
CA19-9 level, normal /high	1.91	0.981–3.735	0.057			
ALRI	3.81	2.130–6.810	0.0001	2.99	1.638–5.459	0.0004

*ALRI* albumin-lymphocyte-RAS index.

### TNM stage in the low-ALRI group and-high-ALRI group

Among the total patient series, 104 patients (23.4%) had been diagnosed with stage I cancer, 184 (41.4%) with stage II, and 157 (68.2%) with stage III. In the high-ALRI group, there were 16 patients with stage I cancer (15.4%), 60 with stage II (32.6%), and 50 with stage III (31.9%). We observed a significant trend in which the number of patients with a low-ALRI value increased as the stage progressed (RFS: p = 0.002) (Table 4).

**Table 4:** Correlation between colorectal cancer stage and the ALRI status.

	Low ALRI	High ALRI	p-value
Stage I: n = 104 (23.4%)	88 (84.6%)	16 (15.4%)	0.002
Stage II: n = 184 (41.4%)	124 (67.4%)	60 (32.6%)	
Stage III: n = 157 (35.3%)	107 (68.2%)	50 (31.9%)	

*ALRI* albumin-lymphocyte-RAS index.

### Kaplan–Meier curve of SIRI in CRC patients

**Figure 2:**
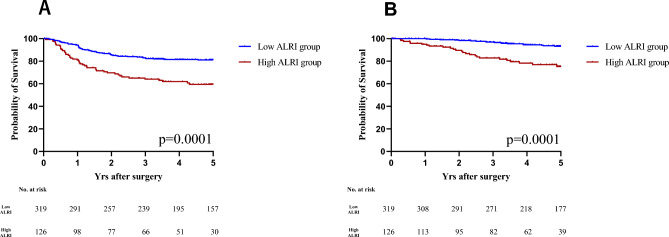
Kaplan–Meier analysis for CRC in all stages according to RFS (**A**) and OS (**B**).

Survival analyses were carried out by comparing the low-ALRI group and high-ALRI group based on the defined cutoff value for ALRI. The Kaplan–Meier curves exhibited significant differences between the two groups for both the 5 year RFS and OS rates (p = 0.0001 for both), suggesting potential prognostic value for SIRI. As illustrated in Fig. 2, the 5 year RFS rates for the high-ALRI and low-ALRI groups were 59.4% and 81.1%, respectively, while their 5 year OS rates were 75.3% and 93.2%, respectively.

**Figure 3:**
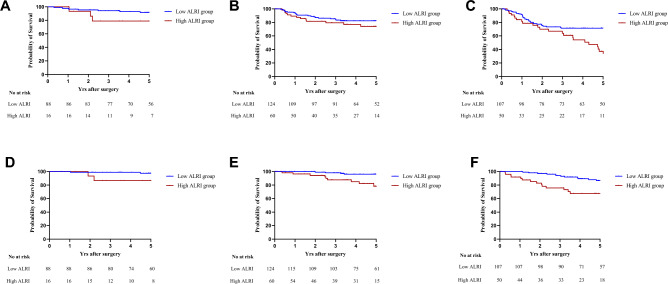
Kaplan–Meier analysis for the RFS of colorectal cancer patients in stratification analysis based on TNM stage: stage I (**A**), stage II (**B**) and stage III (**C**). Kaplan–Meier analysis for the OS of colorectal cancer patients in stratification analysis based on TNM stage: stage I (**D**), stage II (**E**) and stage III (**F**).

Our investigation into the correlation between ALRI levels and tumor nodes metastasis (TNM) staging unveiled a significant association of the high-ALRI group with a poorer prognosis in stage III CRC (p < 0.0001; Fig. 3C). In stage I and II CRC, while there was a tendency towards a poorer prognosis in the high-ALRI group, the difference did not reach statistical significance (p = 0.129, 0.257; Fig. 3A,B). For OS, unveiled a significant association of the high-ALRI group with a poorer prognosis in stage II and III CRC (p = 0.003, 0.002; Fig. 3E,F). In stage I CRC, while there was a tendency towards a poorer prognosis in the high-ALRI group, the difference did not reach statistical significance (p = 0.191; Fig. 3D).

### Prognostic value of ALRI

**Figure 4:**
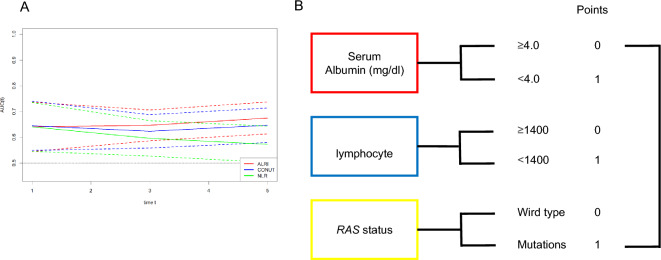
(**A**) Time-dependent ROC curves of ALRI, CONUT, and NLR for prediction of relapse-free survival. The horizontal axis represents year after surgery, and the vertical axis represents the estimated AUC for survival at the time of interest. Dotted lines indicate 95% confidence intervals. *ALRI* albumin-lymphocyte count-RAS index, *CONUT* controlling nutritional status, *NLR* neutrophil-to-lymphocyte ratio. (**B**) Flowchart of the current study. Serum albumin, lymphocyte, *RAS* status.

ALRI was able to predict the outcome of patients after CRC with curative surgery. Comparisons with other prognostic models using nutrition and inflammation are needed to determine how useful they are. Time dependent ROC was used to compare its usefulness with other prognostic models (CONUT and NLR). The results showed that the area under the curve of ALRI was always higher than that of other models. This suggested that ALRI was always more useful than other models (Fig. 4A).

## Discussion

There is growing evidence indicating that, beyond the TNM stage, factors such as nutrition and inflammation play crucial roles in predicting the survival of patients with CRC^11,12^. We created and verified a classification system that incorporates data on cancer genes, supplementing information from nutritional and inflammatory factors. Our developed classification methods have shown strong performance in predicting postoperative outcomes for CRC patients. These user-friendly models are valuable for guiding treatment decisions and follow-up strategies for individuals with CRC.

We used serum albumin, lymphocyte counts, and *RAS* mutations as prognostic factors in this study. Hypoalbuminemia serves as an indicative marker for malnutrition and cachexia, with some studies demonstrating its correlation with adverse outcomes across diverse cancer types^13,15^. Lymphocytes play a crucial role in the host’s immune function, and a reduction in their numbers corresponds to a decline in the host's antitumor immunity, resulting in a poorer prognosis^16,17^. The prognosis prediction using lymphocytes includes the NLR and LMR. It has been reported that each of these is an independent prognostic factor in patients with CRC^6,7^. Upon scrutinizing *RAS* mutants, it was observed that the G12V variant exhibited a diminished guanosine triphosphatase (GTPase) activity, amounting to 25% of the G12D mutant and a mere 10% of the wild-type form^18,19^. Additionally, these *RAS* mutations exhibited a decreased affinity for binding GTPase-activating proteins, further compromising GTPase function. This alteration in functionality modifies the threshold required for triggering cancer apoptosis, potentially amplifying the transformative capabilities of cells and evading apoptosis^20,21^. Consequently, CRC harboring G12V or G12C mutations has been associated with an unfavorable prognosis^4,22^. Recent research has shed light on the significance of regulated necrosis-like cell death, unveiling its diverse physiological and pathological roles along with potential medical applications. Necroptosis and pyroptosis stand out as prominent examples of controlled cell death with necrotic features, including cell swelling, plasma membrane permeabilization, and eventual rupture^23^. These processes were reported that entail the release of damage-associated molecular patterns (DAMPs), such as HMGB1 and inflammatory cytokines like interleukin-1β (IL-1β) and IL-18, upon cell membrane disruption, thus inciting an inflammatory cascade^24^. In the future, genetic mutations and inflammatory makers will become more important.

Exploring the nuanced connections between genetic factors and inflammation has become a focal point in computational biology research. Advancements in techniques, such as deep learning models, enable the identification of intricate interactions among genes and the environment^25,26^. These interactions hold promise for personalized risk assessment, especially concerning conditions influenced by complex inflammatory processes, such as post-surgical outcomes. Recent studies employing these methods highlight their potential in uncovering complex genetic interactions^27^. Furthermore, integrating these findings with future ordinary differential equation models, incorporating specific inflammatory markers identified in this study, could provide a deeper understanding of the dynamic relationship between inflammation and post-surgical outcomes^23,28^. By amalgamating clinical data with theoretical models, we can lay the groundwork for more robust risk prediction models. Ultimately, this approach may lead to the development of personalized preventive and therapeutic strategies.

Within the High-ALRI group, a higher incidence of right-sided CRC cases was observed, along with a greater representation of elderly patients. This observation aligns with a reported trend wherein a comparison of clinicopathological characteristics between right-sided and left-sided CRC revealed a higher prevalence of elderly patients on the right side of the colon^9^. The High-ALRI group exhibited a higher incidence of lymphatic invasion and elevated levels of tumor markers, indicating a greater number of positive cases. This observation suggests that a significant portion of the patients in this group may be experiencing advanced stages of cancer. Further, ALRI demonstrated utility in Stage II and Stage III CRC patients, implying that in Stage I CRC, alterations in albumin and lymphocyte levels might be less pronounced, potentially due to the smaller tumor volume^13^.

Currently, TNM staging is the most commonly employed method for predicting survival outcomes and guiding treatment decisions. However, since TNM staging occurs postoperatively, it cannot predict survival preoperatively or guide further treatment strategies. Additionally, TNM staging solely reflects the tumor's biological behavior. Cancer prognosis is influenced by the host's nutritional status and inflammatory response. ALRI considers lymphocyte, as well as albumin and *RAS* status, offering insights into the tumor microenvironment and the host's preoperative inflammatory response and nutritional status. Our research demonstrates that preoperative ALRI outperforms CONUT and NLR in prognostic discrimination. Thus, utilizing a combination of parameters reflecting both nutritional status, systemic inflammatory status and *RAS* status may be crucial for accurately predicting survival outcomes in CRC patients.

In comparison to current tools addressing immunonutritional interventions, our system stands out due to its superior performance. By integrating oncological, nutritional, and immunological parameters, it surpasses existing nutritional indices in predicting postoperative adverse events. Moreover, our system targets immunonutritional interventions specifically towards patients who stand to gain the most. Our study's findings suggest that proactively managing inflammation and providing nutritional support early on could enhance the prognosis for cancer patients. Identifying patient status before surgery holds various clinical benefits, including prognostic stratification and tailored treatment. Timely detection and improvement of malnutrition and inflammation have the potential to yield improved outcomes for patients^29^.

This study has several limitations that merit consideration. First, the retrospective nature of the study inevitably introduced selection bias, despite the strict adherence to inclusion and exclusion criteria during sample selection. Additionally, the significance of ALRI should be confirmed through validation in other cohorts. Second, the evaluation was based on a relatively small number of patients. Thirdly, confounding factors like infection, ischemia, or acute coronary disease, which could impact serum ALB levels, were not taken into account. Fourthly, the examination of underlying diseases that might influence serum ALB levels, such as liver cirrhosis and chronic renal failure, was not conducted. Fifthly, the optimal cut-off value for the preoperative albumin level and total lymphocyte count remains unknown, despite setting it at 4.0 and 1400 in this study using ROC analysis. Therefore, a large prospective study is warranted to validate and further explore our findings. Sixthly, recent advancements in interaction prediction research across diverse computational biology domains offer valuable insights into genetic markers and their linked diseases. Introducing a novel computational model could enhance the outcomes of our research efforts^30,31^.

In conclusion, this study proposes that preoperative ALRI can function as a straightforward and valuable predictor for gastric cancer prognosis. Furthermore, ALRI can be integrated into preoperative prognosis stratification and postoperative follow-up, contributing to the customization of individualized treatment strategies for CRC.

## Materials and methods

### Patients

We included 445 consecutive patients diagnosed with stage I–III CRC according to the 8th edition of the American Joint Committee on Cancer (AJCC) staging system^14^. These patients underwent curative resection at Teikyo University Hospital in Japan between 2012 and 2017, and surgical procedures for these patients were elective. The study enrolled a total of 445 patients, each of whom provided written informed consent for the utilization of their data. All patient information was de-identified prior to the data analyses to maintain patient anonymity. This study was approved by the Teikyo University Ethics Committee (No. 19-127). The study protocol conforms to the ethical guidelines of the 1975 Declaration of Helsinki and its later amendments.

### Follow up

The surgical resection was deemed curative when there were no indications of tumor recurrence, and the complete histological and macroscopic removal of distant metastases was confirmed. Subsequent to the surgery, patients underwent regular follow-up assessments following a specified schedule. For the initial 3 years, follow-up visits were scheduled every 3 months, followed by visits every 6 months for the subsequent 2 years. At each follow-up, a physical examination was conducted, and the levels of serum tumor markers, such as carcinoembryonic antigen (CEA) and carbohydrate antigen 19-9 (CA19-9), were measured. A colonoscopy examination was performed 1–2 years post-surgery (or annually in the case of rectal cancer). Thoraco-abdominal computed tomography scans were typically carried out every 6 months.

Criteria for CRC recurrence encompassed radiological, clinical, and/or pathological evidence of cancer cells manifesting either locally or in distant locations from their original site. This comprehensive follow-up approach aimed to promptly identify any indications of tumor recurrence or metastasis and initiate appropriate treatment as needed.

### Stational analyses

The χ^2^ test and Fisher’s exact test were used for the comparisons of categorical data, and the Wilcoxon rank-sum test was used for unpaired continuous variables. For paired continuous variables, the Wilcoxon signed-rank test was applied. ROC curves for recurrence were determined, and Youden’s index was used to decide the cut-off values of serum albumin and lymphocyte count.

RFS over a 5 year period was determined as the duration from the date of surgery to the date of either tumor recurrence or death from any cause within 5 years post-surgery. OS was characterized as the time span between the date of surgery and the date of death from any cause within the 5 year post-surgery period.

We conducted a comparative analysis of RFS and OS between the groups, employing Kaplan–Meier curves. The disparity in RFS and OS was assessed using the log-rank test. We then performed univariate analyses by using a Cox-proportional hazards model to investigate the factors which affect RFS and OS, and we subsequently performed a multivariate analysis by a Cox-proportional hazards model using factors with a p < 0.05 in the univariate analysis. Differences with a p value of less than 0.05 were considered significant in all analyses^32^.

### ALRI calculation method

The definition of ALRI was based on the levels of serum albumin, total lymphocyte count, *RAS* status. The Youden index was used to calculate cut-off values for serum albumin and total lymphocyte counts for recurrence, with a score of 1 for serum albumin < 4.0 g/dL and total lymphocyte count < 1400 mg/dL, respectively, and 0 for all other values. One point was given for mutations in *KRAS* G12V and *KRAS* G12C, and zero for *RAS* wild type. The ALRI was defined as the sum of the scores of the above parameters (Fig. 4B). The patients were divided into two groups based on their ALRI: 0, 1 or 2, 3.

### Ethics

This study was approved by the Teikyo University Ethics Committee (No. 19-127).

## Data Availability

The datasets collected in this study can be obtained from the corresponding author upon a reasonable request.
